# Supplementary material for: Treatment effect modification due to comorbidity: Individual participant data meta-analyses of 120 randomised controlled trials
Source: PLoS Med. 2023 Jun 6;20(6):e1004176. doi: 10.1371/journal.pmed.1004176 (PMC10243630; doi:10.1371/journal.pmed.1004176)
Supplement: S1 File — This file contains a more detailed description of the statistical analysis and model specifications. (DOCX) [file pmed.1004176.s001.docx]

Detailed description of modelling

# Continuous outcomes

For each index condition/treatment comparison combination, in separate analyses we examined covariate-treatment interactions for i) age and sex, ii) a comorbidity count, iii) the six commonest conditions (as binary yes/no) for each indication and iv) continuous markers of underlying diseases (body mass index (bmi), estimated glomerular filtration rate (eGFR), Fibrosis 4 score (fib4), haemoglobin (hgb) and mid-blood pressure (mbp)).

All analyses were performed in two stages. First, via individual-level participant data (IPD) analysis within each trial repository. Secondly, as a meta-analyis of the outputs of the IPD analyses.

## Treatment, age and sex

### IPD analysis

For each trial and each outcome, we fitted separate linear regression models to individual-level participant data within the relevant trial repository.

$${final}_{i}={\beta_{0}\cdot base_{i}+ \beta}_{1}+$$

$$\beta_{2}\cdot arm_{i}+\beta_{3}\cdot sex_{i}+\beta_{4}\cdot age_{i}+$$

$$\beta_{5}\cdot\mathrm{arm}_{i}\cdot sex_{i}+\beta_{6}\cdot\mathrm{arm}_{i}\cdot age_{i}$$

Where final and base are the final and initial measure of the outcome of interest, treatment arm and sex are binary variables and age is age rescaled by subtracting 60 from the age (in years) then dividing by 15. Participants are indexed by i. We included the initial measurement among the covariates; this means that the coefficients for the remaining variables are adjusted for pre-treatment response. For each trial and outcome, we exported the resultant model coefficients, standard errors and variance-covariance matrices for subsequent meta-analysis.

### Meta-analysis

For this analysis, three coefficients were of interest:- arm, age-arm interaction and sex-arm interaction. Therefore these three coefficients, and a 3x3 submatrix taken from the IPD-model variance-covariance matrix (above) were modelled as the outcome in a hierarchical Bayesian meta-analysis using a multivariate normal likelihood:-

$$\left[ \begin{matrix} \beta_{2} \\ \beta_{5} \\ \beta_{6} \end{matrix} \right] \sim MVN\left( \left[ \begin{matrix} \theta_{1} \\ \theta_{2} \\ \theta_{3} \end{matrix} \right] , \left[ \begin{matrix} se_{\beta_{2}}^{2} & se_{\beta_{2}}se_{\beta_{5}}\rho_{1} & se_{\beta_{2}}se_{\beta_{6}}\rho_{2} \\ se_{\beta_{5}}se_{\beta_{2}}\rho_{1} & se_{\beta_{5}}^{2} & se_{\beta_{5}}se_{\beta_{6}}\rho_{3} \\ se_{\beta_{6}}se_{\beta_{2}}\rho_{2} & se_{\beta_{6}}se_{\beta_{5}}\rho_{3} & se_{\beta_{6}}^{2} \end{matrix} \right] \right)$$

All of the β’s and θ’s depend on trial (and hence on treatment comparison) but the subscripts have been omitted for clarity.

The linear predictor was:-

$$\theta_{k,j}=\alpha_{k}+trial_{k,j}$$

Where for the k terms of interest from the IPD models (β_2_, β_5_ and β_6_):- α_k_ are the overall effects and trial_k,j_ are the trial-specific effects for the j-trials (as differences from the overall effect). For indications with fewer than five trials, the trial estimate was dropped, reducing the model to a fixed effect analysis.

The trial level effects were assumed to be multivariate normally distributed:-

$$trial_{k,j}\sim MVN\left( 0,V_{trial} \right)$$

As per guidance in the stan manual (https://mc-stan.org/docs/2_29/stan-users-guide/multivariate-hierarchical-priors.html) the covariance matrix (V) was decomposed into a scale s (for each term) and a correlation matrix R. Half normal and Lewandowski-Kurowicka-Joe (LKJ) priors were then placed on these terms respectively:-

$$s_{k,trial}\sim N(mean_{k}=0, sd_{k}=1)$$

$$R_{trial}\sim LKJ(1)$$

The prior for the overall effects $\alpha_{k}$ were assumed to be normally distributed:-

$$\alpha_{k}\sim N\left( mean_{k}=0,sd_{k}=1 \right)$$

## ii) comorbidity count

Analyses for the comorbidity count-treatment interaction were similar except that the IPD analysis included two additional terms – comorbidity count and arm-comorbidity count interaction. Also, as for this analysis we were only interested in the arm-comorbidity count interaction, the multivariate normal distributions (for the likelihood and the random effects) all reduced to normal distributions. As such the linear predictor for comorbidity count was:-

$$\theta_{j}=\alpha+trial_{j}$$

In order to generate an informative prior for conditions/treatment comparisons not included in our data, we also fitted a more complex model including all trials with continuous outcomes, with additional terms to account for the fact that this analysis included treatment comparison and index condition. The results of this model were only used for the results presented in the section headed “Informative priors for subsequent analyses including different index condition/treatment comparisons”.

The linear predictor for this model was:-

$$\theta_{j}=\alpha+trial_{j}+{compar}_{l}+{condition}_{m}$$

where compar_k,l_ was for the treatment comparison level effects (as differences from the overall effect) and condition_k,m_ was for the index condition level effects (as differences from the overall effect).

From this model, we then obtained 1,000 draws from $\alpha$and (for each draw randomly) from the j *trials*, l *compars* and m *conditions* to obtain 1000 predictions (y) for a treatment comparison for an indication not included in the modelling. We then fitted a t-distribution to y.

$$y \sim\text{student-}t(df=d, mean= m, sd=s)$$

d had a gamma (shape = 2, rate = 0.1) prior, m a student-t prior (df = 3, mean = 0, s = 2.5) and s a half-student-t prior (df = 3, mean = 0, s = 2.5).

## iii) Six commonest conditions

The analysis of the covariate-treatment interactions for the six commonest conditions was similar to that for the “treatment, age and sex” except that the IPD analysis model included 12 additional terms. These were 6 terms for each morbidity (coded 0/1) (β_7_ to β_12_) and 6 terms for each arm-comorbidity interaction (β_13_ to β_18_).

The latter 6 coefficients were of interest:- β_13_ to β_18_. Therefore these six coefficients, and a 6x6 submatrix taken from the IPD-model variance-covariance matrix were modelled as the outcome in a hierarchical Bayesian meta-analysis using a multivariate normal likelihood:-

$$\left[ \begin{matrix} \beta_{13} \\ \beta_{14} \\ \beta_{15} \\ \beta_{16} \\ \beta_{17} \\ \beta_{18} \end{matrix} \right] \sim MVN\left( \left[ \begin{matrix} \theta_{1} \\ \theta_{2} \\ \theta_{3} \\ \theta_{4} \\ \theta_{6} \\ \theta_{6} \end{matrix} \right] , \left[ \begin{matrix} se_{\beta_{13}}^{2} & se_{\beta_{13}}se_{\beta_{14}}\rho_{1} & \ldots\\ se_{\beta_{14}}se_{\beta_{13}}\rho_{1} & se_{\beta_{14}}^{2} & \ldots\\ \ldots& \ldots& \ldots\\ \ldots& \ldots& \ldots\\ \ldots& \ldots& \ldots\\ \ldots& \ldots& \ldots\end{matrix} \right] \right)$$

All of the β’s and θ’s depend on trial (and hence on treatment comparison) but the subscripts have been omitted for clarity.

The rest of the meta-analysis was as per the “treatment, age and sex” analysis.

## iv) Continuous markers of possible underlying disease

The analysis of the covariate-treatment interactions for the continuous markers of underlying disease was similar to that for the “treatment, age and sex” except that the IPD analysis model included 10 additional terms. There were 5 terms for each continuous marker -bmi, egfr, fib4, hgb and mbp – (β_7_ to β_11_) and 5 terms for each arm-marker interaction (β_12_ to β_16_).

The latter 5 covariates were of interest:- β_12_ to β_16_. Therefore these six coefficients, and a 5x5 submatrix taken from the IPD-model variance-covariance matrix were modelled as the outcome in a hierarchical Bayesian meta-analysis using a multivariate normal likelihood:-

$$\left( \begin{matrix} \beta_{12} \\ \beta_{13} \\ \beta_{14} \\ \beta_{15} \\ \beta_{16} \end{matrix} \right) \sim MVN\left( \left( \begin{matrix} \theta_{1} \\ \theta_{2} \\ \theta_{3} \\ \theta_{4} \\ \theta_{5} \end{matrix} \right) , \left[ \begin{matrix} se_{\beta_{12}}^{2} & se_{\beta_{12}}se_{\beta_{13}}\rho_{1} & \ldots\\ se_{\beta_{13}}se_{\beta_{12}}\rho_{1} & se_{\beta_{13}}^{2} & \ldots\\ \ldots& \ldots& \ldots\\ \ldots& \ldots& \ldots\\ \ldots& \ldots& \ldots\end{matrix} \right] \right)$$

All of the β’s and θ’s depend on trial (and hence on treatment comparison) but the subscripts have been omitted for clarity.

The rest of the meta-analysis was as per the “treatment, age and sex” analysis.

# Non-continuous outcomes – count and binary outcomes

For trials with non continuous outcome data we only performed an analysis for comorbidity as a count.

The analysis was similar to that described above (Continuous outcomes – ii. comorbidity count) except that the IPD models has a Poisson likelihood, log-link and an offset term for follow-up time (rather than the normal likelihood, identity link and term for the measure at baseline used for continuous outcomes).

For trials with categorical outcome data (eg fracture yes/no), the analysis was also similar. For these indications, the IPD models has a Binomial likelihood and logit-link. For the thromboembolism indication only, we also included a random effect term to reflect more specific indications within the broader indication – primary prevention, secondary prevention, and treatment of thromboembolic disease.
