# Supplementary material for: Treatment effect modification due to comorbidity: Individual participant data meta-analyses of 120 randomised controlled trials
Source: PLoS Med. 2023 Jun 6;20(6):e1004176. doi: 10.1371/journal.pmed.1004176 (PMC10243630; doi:10.1371/journal.pmed.1004176)
Supplement: Attachment — Submitted filename: hanlon.pdf [file pmed.1004176.s005.pdf]

This paper reports a number of individual participant meta–analyses of the effect of comorbidity in clinical trials.

I was asked for a statistical report and I interpret that to include all aspects of the design and conduct of the study.

Points of detail

Page 6 Choosing the most extreme arms could be criticised but since in fact the authors were unable to find evidence even using them then it can be justified.

Page 7 It is not super–clear to me how age enters the models. Do the authors mean they divided age in years by 15 and used it as continuous (which would be fine to avoid excessive zeroes after the decimal point) or do they mean they reduced it to categories with a width of 15 years? Categorising an essentially continuous variable wastes information (Altman and Royston, 2006; Royston et al., 2006) and leads to models which are often implausible as they predict the effect remaining flat within categories and then jumping to a new value at the category boundary. It is, of course, a shame we do not have a common word in English for a period of 15 years akin to decade for ten (well we do have indiction `https://en.wikipedia.org/wiki/Indiction`) so the authors could speak of measuring age as so many indictions but that is liable to lead to incomprehension.

Page 8 Choosing between fixed and random effects based on the number of studies is an unusual choice. I assume this is motivated by the lack of precision in the estimate of τ^2 when the number of trials is small but is switching to fixed effects the answer to that fact? I am persuaded by Rice et al. (2018) and previous authors that we should decide based on the underlying scientific model.

Page 8 Good to see the package cited but something seems to have gone adrift with the referencing here as reference 29 is not, as far as I can see, a citation to package brms. Strangely enough 29 is not a superscript like all the other citations.

Page 10 There does not seem to be any parallel comment on the evidence for effects of sex.

Page 12 I applaud the decisions to give us all the forest plots and not just overall effects but presenting these over two pages makes it a real struggle to see what is going on because everyhting is so tiny. It was some

time before I realised that each row contained two pieces of information. There is not, as far as I know, a limitation on the number of figures so I would split this up over more pages and possibly more figures. I would also suggest Figure 2c might be split off into a separate figure to emphasise it is single trial data not meta–analytic.

Page 16 Table 3 had me baffled. Why are there two rows for primary prevention of bleeding? Incidentally I assume that a similar process to that outlined on page 7 was adopted to reverse the direction of scoring as I would have thought DVT/PE and bleeding were opposite effects. Bear in mind though I am not a clinician. We do not seem to get forest plots here which is a shame.

Page 17 Perhaps worth mentioning that not only does the absence of high numbers of comorbidities affect generalisability but restricting the range makes it more difficult to find evidence of effect.

Page 20 Good to give us the code but I get a 404 when I click on the link in the paper.

Summary

No major issues.

Michael Dewey

References

D G Altman and P Royston. The cost of dichotomising continuous variables. *British Medical Journal*, 332:1080, 2006.

K Rice, J P T Higgins, and T Lumley. A re–evaluation of fixed effect(s) meta–analysis. *Journal of the Royal Statistical Society*, 181:205–227, 2018.

P Royston, D G Altman, and W Sauerbrei. Dichotomizing continuous predictors in multiple regression: a bad idea. *Statistics in Medicine*, 25:127–141, 2006.
