# Supplementary material for: Treatment effect modification due to comorbidity: Individual participant data meta-analyses of 120 randomised controlled trials
Source: PLoS Med. 2023 Jun 6;20(6):e1004176. doi: 10.1371/journal.pmed.1004176 (PMC10243630; doi:10.1371/journal.pmed.1004176)
Supplement: Attachment — Submitted filename: Assessing treatment effect modification due to comorbidity - PLoS One - review.pdf [file pmed.1004176.s006.pdf]

**Assessing treatment effect modification due to comorbidity using individual participant data from industry-sponsored drug trials**

Dr Peter Hanlon and colleagues for PLOS Medicine

This is paper addresses an important topic – evidence on treatment efficacy for patients with multiple comorbid conditions. Currently, there is absolutely nothing in the literature that addresses this issue. Hence this paper is a first of its kind. While I am very enthusiastic and supportive of the paper, I have major concerns on some of the statistical methodology aspects.

1. The paper doesn't provide any descriptive information on the distribution of sample size, age, sex, and comorbidities in the trials. A concise summary would be essential for the main paper, and additionally a more detailed description would be useful as a Supplement.
2. The main conclusion of the paper is that there is no substantial variation in treatment effect in terms of comorbidity, i.e., the treatment*comorbidity interaction is not statistically significant. I am concerned about the validity of this result. It is quite likely that none of the trials had adequate number of patients with 3 or more comorbidities. Then, how can one rely upon a meta-analytic conclusion that is derived from weakly designed, under-powered, individual studies? Combining a large number of weak and highly variable studies is unlikely to provide valid and meaningful results. The paper should acknowledge this fundamental limitation.
3. What is the outcome model in the treatment*comorbidity interaction problem, within each trial? Does it also include age and sex interactions with the treatment? Can you write it out?
4. I have a major concern with the manner in which treatment*comorbidity-count is modeled as a linear term, i.e. the impact of one-unit increase in comorbidity is the same whether it goes from 0 to 1 or from 5 to 6. This is a highly untenable assumption. I would recommend that the authors do another set of analyses where this variable is treated as a binary covariate: 2 or less versus 3 or more.
5. Were treatment*age and treatment*sex interactions included in the model where treatment*comorbidity was the focus?
6. I was wondering why the main effect for treatment ("arm") was not included in the meta-analysis of treatment*comorbidity interaction, while it was included in the meta-analysis of treatment*age and treatment*sex interactions. I would argue that the main effect ought to be included.
7. I would also suggest sensitivity analyses for the prior parameters of the variance parameters: standard deviation of half-normal and the LKJ parameter for the correlation matrix. The paper should document how decreasing or increasing these parameters impacted the final meta-analytic estimates of the interaction effects.
8. I did not understand the logic behind generating an informative prior for conditions/treatment comparisons not included in our data. Can this be better explained?
9. The authors state the following: "*We included the initial measurement among the covariates; this means that the coefficients for the remaining variables are interpretable as estimates for the change in the outcome measurement (ie final – base) per unit increment in the covariate*."
   This interpretation of the coefficients is not quite correct. It is the expected change in post-treatment response conditional on the pre-treatment response. It cannot be interpreted as

estimate of pre-post change for which one would have to model the conditional expectation of the change itself: E[final - base | covariates]
