# Supplementary material for: Treatment effect modification due to comorbidity: Individual participant data meta-analyses of 120 randomised controlled trials
Source: PLoS Med. 2023 Jun 6;20(6):e1004176. doi: 10.1371/journal.pmed.1004176 (PMC10243630; doi:10.1371/journal.pmed.1004176)
Supplement: Attachment — Submitted filename: Response to reviewers.docx [file pmed.1004176.s008.docx]

Thank you for the opportunity to submit a revised version of our manuscript. Please see below our responses to all editorial and reviewer comments along with detail of our responses to each.

Yours sincerely

Dr Peter Hanlon, on behalf of the study authors

Editor comment:

GENERAL

Please respond to all editor and reviewer requests detailed below in full.

Please include line numbers in your revised version starting at 1 and in continuous sequence thereafter.

Author response:

We have added line numbers as requested.

Editor comment:

TITLE

Please revise your title according to PLOS Medicine's style. Your title must be nondeclarative and not a question. It should begin with main concept if possible. "Effect of" should be used only if causality can be inferred, i.e., for an RCT. Please place the study design ("A randomized controlled trial," "A retrospective study," "A modelling study," etc.) in the subtitle (ie, after a colon).

Author response:

We have revised the title to “Treatment effect modification due to comorbidity: individual participant data meta-analyses of industry-sponsored randomised controlled trials” (page 1)

Editor comment:

ABSTRACT

Please structure your abstract using the PLOS Medicine headings (Background, Methods and Findings, Conclusions).

Please combine the Methods and Findings sections into one section, “Methods and findings”.

Author response:

Thank you, we have changed this as requested.

Editor comment:

Abstract Methods and Findings:

Please include additional details regarding the study population - number of participants, age ranges etc, and the setting – region/country etc., years during which the studies took place, length of follow up, and main outcome measures.

Author response:

We have added the following detail to the abstract methods and findings section:

“We obtained individual participant data for 120 industry-sponsored phase 3/4 trials across 23 index conditions (n=128,331). Trials had to be registered between 1990 and 2017 and have recruited ≥300 people. Included trials were multicentre and international. For each index condition, we analysed the outcome most frequently reported in the included trials.” Page 2 lines 30-33

And

“Mean age in the trials ranged from 37.1 (allergic rhinitis trials) to 73.0 (dementia trials) and percentage of male participants range from 4.4% (osteoporosis trials) to 100% (benign prostatic hypertrophy trials). The percentage of participants with three of more comorbidities ranged from 2.3% (allergic rhinitis trials) to 57% (systemic lupus erythematosus trials).” Page 2 lines 42-45

Editor comment:

Paragraph 1: “…markers of underlying conditions (e.g., estimated glomerular function).” Do you mean estimated glomerular filtration rate (eGFR) to monitor renal impairment? (glomerular “function” can be measured in different ways – filtration rate or urinary protein excretion)

Author response:

Thank you for highlighting this. We have amended to estimated glomerular filtration rate.

 Editor comment:

Some statements might benefit from further quantification with numerical values and statistical information, for example, paragraph 2 line 1 – how many is “few”

Author response:

We have now provided summary details of the included trials (with numerical values) as detailed in the comment above. The sentence highlighted in the comment has been removed in favour of summary data on mean comorbidity quoted above.

Editor comment:

Paragraph 2 line 2 – as above, would it be helpful to support this statement with statistical information Paragraph 2 line 6 – Please revise to “Sodium-glucose co-transporter-2 (SGLT2) inhibitors”

Author response:

We have edited as suggested.

Editor comment:

PLOS medicine requires that the main results are quantified with 95% CIs as well as p values. When reporting p values please report as p<0.001 or where higher as p=0.002 (not <.001 or p=.002).  If not including p values, for the purpose of transparent data reporting, please clearly state the reasons why not.

Author response:

We have not reported p-values as all findings were obtained using Bayesian methods whereas P-values relate to null hypothesis significance testing which is a feature of frequentist (rather than Bayesian) statistics. We therefore present 95% credible intervals for each result, but have not presented p-values.

Editor comment:

Please ensure that any/all numbers presented in the abstract are present and identical to those presented in the main manuscript text.

Author response:

We have checked for discrepancies and confirm that the abstract matches the main text.

Editor comment:

Please include any important dependent variables that are adjusted for in the analyses

Author response:

We have added: “we modelled the interaction between comorbidity and treatment arm adjusted for age and sex.” Page 2 lines 34-35

Editor comment:

In the last sentence of the Abstract Methods and Findings section, please describe the main limitation(s) of the study's methodology.

Author response:

We have added: “The main limitation is that these trial were not designed or powered to assess variation in treatment effect by comorbidity, and relatively few trial participants had >3 comorbidities.” Page 2 lines 53-55

Editor comment:

Abstract Conclusions:

Please emphasize what is new without overstating your conclusions “Our findings support the assumption that estimates of treatment efficacy are constant…” it would be helpful to include a brief justification/explanation of how this helps/what it adds in real terms

Author response:

We have edited the conclusion of the abstract to the following:

“Assessments of treatment effect modification rarely consider comorbidity. Our findings demonstrate that for trials included in this analysis, there was no empirical evidence of treatment effect modification by comorbidity. The standard assumption used in evidence syntheses is that efficacy is constant across subgroups, although this is often criticised. Our findings suggest that for modest levels of comorbidities, this assumption is reasonable. Thus trial efficacy findings can be combined with data on natural history and competing risks to assess the likely overall benefit of treatments in the context of comorbidity.” Page 2 lines 57-63

Editor comment:

AUTHOR SUMMARY

At this stage, we ask that you include a short, non-technical Author Summary of your research to make findings accessible to a wide audience that includes both scientists and non-scientists. The Author Summary should immediately follow the Abstract in your revised manuscript. This text is subject to editorial change and should be distinct from the scientific abstract.

We suggest reviewing our website and published articles for examples <https://journals.plos.org/plosmedicine/>

Please see our author guidelines for more information: <https://journals.plos.org/plosmedicine/s/revising-your-manuscript#loc-author-summary>

Author response:

We have added an author summary as follows:

Author Summary

Why was this study done?

• There is often uncertainty about how treatments for single conditions should be applied to people with two or more long-term conditions (multimorbidity).

• People with multimorbidity are under-represented in randomised controlled trials, however trials rarely report whether the efficacy of treatment differs by the number of additional long-term conditions (comorbidities) or in the presence of specific comorbidities.

What did the researchers do and find?

• We analysed individual-participant data from 120 randomised controlled trials including 128,331 participants across 23 index conditions to assess whether the efficacy of treatment differed depending on the number of comorbidities, or in the presence of any of the most common comorbidities.

• We found no evidence that treatment efficacy differed depending on the number of comorbidities, or by any specific comorbidities, for any of the index conditions and treatment comparisons included in this analysis.

What do these findings mean?

• Within the range of comorbidities included within these trials, treatment effects did not vary by comorbidity. These findings can be used within evidence syntheses to estimate the likely overall benefit of treatments in the context of multimorbidity.

• These findings are limited by the fact that people with multiple comorbidities are under-represented in trials, and those with the highest degree of comorbidity are often excluded.

Page 3

Editor comment:

INTRODUCTION

Paragraph 1 sentence 2: suggest removing “The prevalence of multimorbidity is such that” and starting the sentence with “Most people…”

Please see reviewer 2 comments also

Author response:

Thank you, we have made this edit and agree this improves the sentence in question.

Editor comment:

METHODS and RESULTS

Please include additional regarding the trials and the participants, for example - participants/sample size and dates of recruitment of participants to the trials, length of follow-up, comorbidities.  See also reviewer 3 comments Page 8 para 2: “…brms package.(29)…” what does this numerical refer to?

Author response:

We have added additional detail to table 1 to summarise the age, sex and comorbidity distribution of the included trials. Further detail on each of the individual trials is included in our ‘baseline’ paper (with trial-level detail in the supplementary material) and we now signpost to this detailed data within the footnote of table 1.

Editor comment:

PLOS medicine requires that the main results are quantified with 95% CIs as well as p values. When reporting p values please report as p<0.001 or where higher as p=0.002 (not <.001 or p=.002).  If not including p values, for the purpose of transparent data reporting, please clearly state the reasons why not.

Author response:

We have not reported p-values as all findings were obtained using Bayesian methods whereas P-values relate to null hypothesis significance testing which is a feature of frequentist (rather than Bayesian) statistics. We therefore present 95% credible intervals for each result, but have not presented p-values.

On the other hand, in our Github repository we have provided we data, code and 4,000 samples from the posterior, which means that the entire posterior distribution can be plotted, and almost any summary can be extracted.

We have added a sentence to the methods to justify this in keeping with the editor’s comment on transparent data reporting.

“P-values were not presented as this was a Bayesian analysis. The full posteriors are provided in the project github repository” Page 8 lines 265-267

Editor comment:

FIGURES

Please ensure that all figures are associated with an appropriate caption which clearly describes the figure content without the need to refer to the text. Please ensure all abbreviations are defined within (e.g., Figure 1 - CSDR, YODA, IPD)

Author response:

We have added captions to each of the figures and include definitions of all abbreviations within these.

Editor comment:

Figure 2a, b, c – the plots are very small and not easy to read, including the text. Please revise accordingly. Please ensure that an appropriate legend is included which clearly details what the different color bars and dots mean.  Please define all abbreviations (e.g., IBD). Please see reviewer 1 comments also.

Author response:

Thank you. We have now split figure 2a and 2b across multiple figures to allow these to be larger and in higher resolution. Figure 2c is now presented separately as figure 3 as it concerns single trials (as suggested by reviewer 1).

Editor comment:

TABLES

Please ensure that all tables are associated with an appropriate caption which clearly describes the table contents without the need to refer to the text.  Please ensure that all abbreviations used in the tables are defined in the caption.  For example, 2nd column (outcomes) in table 1, 2nd column table 2 “ACE”

Author response:

Thank you. We have added captions to all tables and included abbreviation definitions within each of these.

Editor comment:

Table 2: would be more accessible if the CIs were placed on the same row. Please revise. Suggest reducing the size of column 2 and considering the use of commas to separate upper and lower bounds in place of the word “to”

Author response:

Thank you for this suggestion and we have revised table 2 as suggested.

Editor comment:

Table 2 legend/caption: refers the reader to table 1 for abbreviations but I could not see that nay have been defined.  In addition, please ensure that each table has its own caption with all abbreviations clearly defined (even if that means repeating them).

Author response:

Thank you for pointing this out. The reference to abbreviations in table 1 was a reference to a previous version of the table which we neglected to delete. We have amended the legend and ensure that all abbreviations within the table are defined in the legend.

Editor comment:

Table 3: This table is rather confusing.  As presented it would suggest that anti-coags listed here are taken to prevent bleeding.  This would not be the case, obviously.  Column 2 header “condition” doesn’t seem appropriate as secondary prevention & acute treatment are not conditions – secondary prevention/acute treatment of what?  Are there 2 rows for bleeding/primary prevention because these investigated different classes of LMWH (B01AB Vs B01AA03)? Please also see reviewer 1 comments. Please revise this table.

Author response:

We have revised and re-structured table 3 to make the presentation of these data clearer.

We have also added the following text to the methods to explain the inclusion of bleeding outcomes (and of 2 separate outcomes) for the analysis of trials of anticoagulation. This information is also now included in the footnote to table 3.:

“For trials of anticoagulants, in addition to the efficacy outcome, we also analysed bleeding events as these are a common and clinically important adverse outcome.” Page 7 lines 218-219

Editor comment:

Please also revise the presentation of the 95% CIs in this table to ensure consistency with other tables.  Suggest the use of commas rather than hyphens, as these can be confused with the reporting of negative values, to separate upper and lower bounds. Please ensure that words are not split across lines. As above please ensure an appropriate caption includes all abbreviations (e.g., DVT, PE).

Author response:

Thank you. We have made these changes.

Editor comment:

DISCUSSION

Please remove all sub-headings from the discussion such that it reads as a single piece of continuous prose, ending in a one paragraph conclusion.  Please present and organize the Discussion as follows: a short, clear summary of the article's findings; what the study adds to existing research and where and why the results may differ from previous research; strengths and limitations of the study; implications and next steps for research, clinical practice, and/or public policy; one-paragraph conclusion.

Author response:

We have re-ordered the discussion as suggested and removed the subheadings as requested. We have added a linking sentence after the limitations and before the implications to highlight the transition:

“Our findings have implications for the conduct of future evidence syntheses.” Page 18 line 470

Editor comment:

Please remove the funding and data availability statements for the end of the main manuscript and include only in the manuscript submission form.

Author response:

We have removed this as requested.

Editor comment:

Please move the ethics statement to an appropriate part of the methods section.

Author response:

We have moved this accordingly.

Editor comment:

REFERENCES

Please ensure that for in-text reference callouts, citations are placed in square parentheses and preceding punctuation, as follows: “…is a global clinical and public health priority [1,2].” Please check and amend throughout. Please note the absence of spaces between citations.

In your bibliography, please ensure that up to but no more than 6 author names are listed, followed by et al., if more than 6 authors contribute to an individual study.

Please ensure that journal names abbreviations are those found in the National Center for Biotechnology Information (NCBI) databases

Author response:

We have edited the references to fit with the PLoS Medicine style as requested.

Editor comment:

SUPPORTING INFORMATION

Please ensure that it is clearly defined for the reader what the data in supplementary tables 1 and 2 refer to.  Please include an appropriate title and caption describing their contents.

Author response:

We have added legends for these files to the manuscript document (Page 21)

Comments from the reviewers:

Reviewer #1:

Reviewer comment:

This paper reports a number of individual participant meta-analyses of the effect of comorbidity in clinical trials. I was asked for a statistical report and I interpret that to include all aspects of the design and conduct of the study.

Points of detail

Page 6 Choosing the most extreme arms could be criticised but since in fact the authors were unable to find evidence even using them then it can be justified.

Author response:

Thank-you. We agree that had we found evidence of HTE by comorbidity it would have been useful to examine other arm comparisons in addition to the most extreme comparison.

Reviewer comment:

Page 7 It is not super clear to me how age enters the models. Do the authors mean they divided age in years by 15 and used it as continuous (which would be fine to avoid excessive zeroes after the decimal point) or do they mean they reduced it to categories with a width of 15 years? Categorising an essentially continuous variable wastes information (Altman and Royston, 2006; Royston et al., 2006) and leads to models which are often implausible as they predict the effect remaining at within categories and then jumping to a new value at the category boundary. It is, of course, a shame we do not have a common word in English for a period of 15 years akin to decade for ten (well we do have indiction https://en.wikipedia.org/wiki/Indiction) so the authors could speak of measuring age as so many indictions but that is liable to lead to incomprehension.

Author response:

We apologise for the ambiguity. We modelled age as a continuous variable (as the reviewer favours), dividing by 15 years so as to scale the coefficients as this helps with model fitting within Stan:

“age (modelled as a continuous variable scaled to 15-year increments, which was close to the standard deviation for most trials),” page 7 lines 230-231

Reviewer comment:

Page 8 Choosing between fixed and random effects based on the number of studies is an unusual choice. I assume this is motivated by the lack of precision in the estimate of _ 2 when the number of trials is small but is switching to fixed effects the answer to that fact? I am persuaded by Rice et al. (2018) and previous authors that we should decide based

on the underlying scientific model.

Author response:

The reviewer is correct that we were concerned that imprecise estimates of the between trial variation was the motivation for using fixed effects. We decided to do so rather than inflating the uncertainty by either putting a highly-influential prior on the between studies standard deviation or assuming a value for the between studies standard deviation for which we have no evidence. However, we agree that many would argue it is more valid to fit a random effects model with a parameter for the between-trial variation, and we agree that there are arguments in favour of this approach. We therefore now also present results random effects results for those meta-analyses where we previously presented fixed effects analyses (ie those with 2-4 trials) using the same model specification we used when there were 5 or more trials. The point estimates were similar in this additional analysis, but with wider credible intervals. We have added the following text to the results:-

“In a sensitivity analysis, rather than using a fixed effects model for meta-analyses where there were fewer than 5 trials, we fit a random effects model. The 95% credible intervals were wider, but the results of these models were otherwise similar to those presented in the main analysis (S3 Table).” Page 14 lines 374-376

Reviewer comment:

Page 8 Good to see the package cited but something seems to have gone adrift with the referencing here as reference 29 is not, as far as I can see, a citation to package brms. Strangely enough 29 is not a superscript like all the other citations.

Author response:

Thank you. This is now cited.

Reviewer comment:

Page 10 There does not seem to be any parallel comment on the evidence for effects of sex.

Author response:

Thank you for highlighting this. We have added the following text:

“Similarly, most interaction terms for sex included the null, with a few exceptions (table 2). For example, for Glucagon-like peptide-1 (GLP-1) analogues the interaction term for sex was 0.29 (0.12 to 0.49) indicating that the lowering effect on HbA1c of GLP-1 analogues is 1.16 (0.48 to 1.96) mmol/mol smaller in men than in women.” Page 12 lines 314-317

Reviewer comment:

Page 12 I applaud the decisions to give us all the forest plots and not just overall effects but presenting these over two pages makes it a real struggle to see what is going on because everyhting is so tiny. It was some time before I realised that each row contained two pieces of information.

There is not, as far as I know, a limitation on the number of figures so I would split this up over more pages and possibly more figures. I would also suggest Figure 2c might be split off into a separate figure to emphasise it is single trial data not meta-analytic.

Author response:

Thank you. We have revised the figures in light of these comments. We have split the presentation of the meta-analyses (originally 2a and 2b) across 5 figures, allowing the text to be larger and better resolution. We have also separated figure 2c into a separate figure pertaining to individual trial results as suggested by the reviewer.

Reviewer comment:

Page 16 Table 3 had me baffled. Why are there two rows for primary prevention of bleeding? Incidentally I assume that a similar process to that outlined on page 7 was adopted to reverse the direction of scoring as I would have thought DVT/PE and bleeding were opposite effects. Bear in mind though I am not a clinician. We do not seem to get forest plots here which is a shame.

Author response:

We apologise for the confusion in Table 3. This was an error made when we changed the values for the different categories (which were abbreviations) to full labels. We have corrected this error. We have provided the trial-level estimates in the github repository which we now highlight in the footnote of the table.

We included two outcomes (DVT/PE and bleeding) for the thromboembolic trials only. We did so because both outcomes are quite common in trials of drugs acting on the clotting/bleeding systems (eg antiplatelets, anticoagulants etc). Unlike for the continuous outcomes, we didn’t change the outcome status (ie coded as 1 for outcome present for both). We hope the tables look clearer now. We have added a short explanation about the analysis of two separate outcomes for these trials to the methods section and to the footnote of table 3.

Reviewer comment:

Page 17 Perhaps worth mentioning that not only does the absence of high numbers of comorbidities affect generalisability but restricting the range makes it more difficult to find evidence of effect.

Author response:

We agree with the reviewer and have added the following text to the limitations:

“… the higher levels of comorbidity observed in clinical practice (e.g. 5 or more comorbidities) are rare within the trial participants. This reduces the likelihood of detecting a comorbidity-treatment interaction were one to exist. Therefore, while our results are consistent with there being no comorbidity-treatment interactions, this should be interpreted within the range of comorbidities, index conditions and treatment comparisons that are presented.” Page 17 lines 444-449

Reviewer comment:

Page 20 Good to give us the code but I get a 404 when I click on the link in the paper.

Author response:

We apologise for this. We have now made the repository publicly available:

<https://github.com/ChronicDiseaseEpi/hte_cm_public>

and have created a doi of the final version which is linked in the paper:

<https://zenodo.org/badge/latestdoi/611754942>

Reviewer comment:

Reviewer #2: This manuscript aimed to explore the impact of comorbidity on relative treatment effect in trials. This is an interesting topic. One strength of this study is that it included trials with individual patients data, and thus the assessment of effect modification is based on within-trial comparisons. However, there are several issues for the authors to address.

1. Abstract

For analysis, authors stated only they performed two-stage IPD meta-analysis. We suggest to add more details, e.g., first conducted regression analysis within trials and then combine interaction coefficients across trials for separate conditions and comparisons.

Author response:

Thank you for this suggestion. We have added the following text to the abstract:

“First, for each trial, we modelled the interaction between comorbidity and treatment arm for the selected outcome. Second, for each treatment within each index condition, we meta-analysed the comorbidity-treatment interaction terms from these trial level models.” Page 2 lines 34-37

Reviewer comment:

2. Abstract

In the conclusion, authors said "Our findings support the assumption that estimates of treatment efficacy are constant". We suggest to highlight that this study is about relative effect (authors have thoughtfully and appropriately discussed relative versus absolute effect in discussion), that is, this study found no evidence of relative effect differences associated with comorbidity.

Author response:

Thank you for highlighting this section. We have added the following text to clarify the scale on which we analysed the outcomes.

“Treatment effects were modelled on the usual scale for the type of outcome (absolute scale for numerical outcomes, relative scale for binary outcomes).” Page 2 lines 40-42

Reviewer comment:

3. Introduction

In the third paragraph, authors discussed limitations of subgroup analysis in meta-analysis. The authors are correct when they state: "trials rarely report subgroup effects by comorbidity, and those that do may be subject to publication bias." However, what systematic review authors do under these circumstances is utilize between-study comparisons that provide much less compelling evidence.  It may be - we think it would be - worth pointing this out before moving on the merits of IPDMA. The within- versus between difference is highlighted in the first structured instrument for addressing subgroup analyses <https://www.cmaj.ca/content/192/32/E901>.

Author response:

We agree with the reviewer and have added the following text:

“… In such circumstances, any assessment of the effect of comorbidity is therefore based on between-trial comparisons which are prone to bias.” Page 4 lines 116-118

Reviewer comment:

4. Introduction

In the last paragraph, for objective of this study, authors mentioned age and sex. Although they added age and sex in the regression model, seems their primary objective is to explore effect modification by comorbidity. Authors may want to remove age and sex from the objective.

Author response:

We agree that the effect of comorbidity is the main focus and aim of the paper and have edited the final paragraph of the introduction accordingly.

Reviewer comment:

5. Methods

We suggest making it clearer about at what level (individual patients, or trials), authors extracted what data and conducted what analysis.

Author response:

We have added an explanation of the levels of each analysis to the footnote of Figure 1:

“**Fig 1: Overview of analysis.** This figure gives an overview of the analysis structure and hierarchy. Analyses of individual-level data within each trial were conducted within two secure repositories (Yale Open Data Access [YODA] shown in pink, and Clinical Study Data Request [CSDR] shown in orange). For each trial, a summary of the results was exported and meta-analysed within each treatment indication (green).” Page 5 lines 148-152

Reviewer comment:

6. Results

To convert the measures into a similar scale, authors divided the estimates by the minimally important difference; however, this makes the interaction coefficients are difficult to interpret. We suggest adding how one should interpret the interaction coefficients for comorbidities (as they did for age treatment interactions).

Author response:

Thank you for highlighting the need for clarity here. We agree that there is a need to spell out how these coefficients should be interpreted, as we do for age an have added text for sex:

“…in the diabetes trials, there appeared to be an attenuation in the treatment effect with increasing age for three drug classes (0.07 (95% CI 0.00 to 0.13) for Sulfonylureas vs SGLT2 inhibitors, 0.09 (95% CI 0.01 to 0.17) for DPP-4 inhibitors vs SGLT2 inhibitors and 0.07 (95% CI 0.04 to 0.11) for SGLT2 inhibitors versus placebo). Taking SGLT2 inhibitors versus placebo as an example, this can be read as follows – ‘the lowering effect on HbA1c of SGLT2 inhibitors versus placebo is 0.28 (95% CI 0.16 to 0.44) mmol/mol smaller (since the MCID for HbA1c is 4 mmol/mol) per 15-year increment in age, for age 50 years versus age 80 years this corresponds to the effect being 0.56 (95% CI 0.32 to 0.88) mmol/mol smaller. Similarly, most interaction terms for sex included the null, with a few exceptions (table 2). For example, for Glucagon-like peptide-1 (GLP-1) analogues the interaction term for sex was 0.29 (0.12 to 0.49) indicating that the lowering effect on HbA1c of GLP-1 analogues is 1.16 (0.48 to 1.96) mmol/mol smaller in men than in women.” Page 12 lines 307-317

We have not presented text in the same way for comorbidities as such a presentation may imply the presence of an interaction where there was no statistical evidence of one. We have therefore added the following text to explain the interpretation of to coefficients for comorbidity.

“This suggests that it is plausible, for all treatments and in all index conditions, that there is no difference in treatment effects by comorbidity (on the absolute scale) within the range of comorbidities observed.” Page 13 lines 333-335

Reviewer comment:

7. Results

In addition to figures, better to also have tables containing the comorbidity treatment interaction coefficients, and measure of inconsistency e.g., I2 (whether interaction coefficients differ across trials). If the authors feel this is too burdensome for the main text such tables could be added in supplementary material.

Author response:

We agree with the reviewer that all data should be provided for transparency. Given the large number of trials and large amount of data, we have provided these data as supplementary data. This includes all trial level interaction coefficients. We have not produced an I^2^ value (as this is typically used in frequentist rather than Bayesian meta-analyses), but do provide samples from the posteriors for all parameters, which fully captures the model outputs (https://zenodo.org/badge/latestdoi/611754942).

Reviewer comment:

8. In relation to other work the authors should discuss another study addressing subgroup effects in IPDMA

<https://academic.oup.com/ije/article/48/2/596/5184552>

These authors found more interactions than the current authors in a sample of IPDMA's, a couple of which were related to comorbidity. It seems to us that the results could be interpreted as essentially consistent with the current results, or somewhat inconsistent. We would be interested in the authors' views.

Author response:

Thank you for highlighting this interesting reference which was interested in claims of clinical relevance for IPDMA reporting statistically significant subgroups. Unfortunately, the focus of the paper this meant that the authors only extracted data for reports of statistically significant subgroups; also even among these the subgroup variables were not systematically characterised (around 25% of subgroups were classified as other). Indeed, from the presented data, diabetes is the only comorbidity which we could reliably identify. 7 statistically significant subgroup variables were of diabetes. It appears that most of these were from the same 27 trial 160,000 participant IPDMA designed to study whether treatment effects differ by diabetes. We were not previously aware of this IPDMA and have now added it to our discussion of the existing literature on studies of heterogeneity of treatment effects by comorbidity.

“Several previous studies have reported findings on treatment effect modification in IPD meta-analyses and meta-analyses of reported subgroup effects. However, these have largely been confined to major cardiovascular disease trials (e.g., for showing similar efficacy of statin in people with and without diabetes [26], differential benefit of blood pressure lowering therapy in people with and without diabetes [27], or showing questionable net benefit of aspirin in primary prevention [28]) or to concordant conditions defined as those closely related to the index condition or target event for the trial (such as hypertension in stroke trials [29]).” Page 16 lines 402-408

Reviewer comment:

9. The authors deal well with the issue of multimorbidity and baseline risk. Another key contextual issue they may want to address is the burden of treatment in those with multi-morbidity who are generally on a number of medications whenever they consider adding another.  Looking at some of the literature on minimally disruptive medicine may be useful in this regard. <https://pubmed.ncbi.nlm.nih.gov/27417747/>

Author response:

We agree, and have edited to the following (added text in bold):

“Although we found that relative treatment efficacy did not differ by comorbidity count, net overall treatment benefits may nonetheless differ in people with differing degrees of comorbidity. This is because differences in the baseline risk (e.g., the absolute risk of the outcome that the treatment is intended to prevent), differences in susceptibility to treatment-related adverse events, differences in competing risks (e.g., absolute risk of mortality from other causes), **and difference in the burden of treatment (e.g. higher treatment burden in the context of multimorbidity leading to reduced concordance or reduced quality of life)** may all lead to differences in the net overall benefit of treatment.” New text on page 16 lines 425-427

Reviewer comment:

Reviewer #3: Please see the attached PDF for my comments to the authors.

 This is paper addresses an important topic – evidence on treatment efficacy for patients with multiple comorbid conditions. Currently, there is absolutely nothing in the literature that addresses this issue. Hence this paper is a first of its kind. While I am very enthusiastic and supportive of the paper, I have major concerns on some of the statistical methodology aspects.

1. The paper doesn’t provide any descriptive information on the distribution of sample size, age, sex, and comorbidities in the trials. A concise summary would be essential for the main paper, and additionally a more detailed description would be useful as a Supplement.

Author response:

Thank you for making this suggestion. We had initially referred to our detailed “baseline” paper where we compare comorbidity counts in clinical trials with those in the community, but agree that summary information should also be included here. We have added this to table 1.

Reviewer comment:

2. The main conclusion of the paper is that there is no substantial variation in treatment effect in terms of comorbidity, i.e., the treatment*comorbidity interaction is not statistically significant. I am concerned about the validity of this result. It is quite likely that none of the trials had adequate number of patients with 3 or more comorbidities. Then, how can one rely upon a meta-analytic conclusion that is derived from weakly designed, under-powered, individual studies? Combining a large number of weak and highly variable studies is unlikely to provide valid and meaningful results. The paper should acknowledge this fundamental limitation.

Author response:

As can be seen in Table 1 the percentage of patients with 3 or more comorbidities ranged from around 2% (for a trial in allergic rhinitis) to around 50% (eg for trials in osteoporosis). We apologise that this information was not presented before, but hope that this provides reassurance that the individual trials contribute useful information to the meta-analysis. Moreover, the credible confidence intervals are precise, indicating that moderate or large comorbidity count-treatment interactions are unlikely.

Nonetheless, we agree with the reviewer with respect to the trials being neither designed nor powered to assess comorbidity-treatment interactions. We therefore have added the following text to the abstract:

“The main limitation is that these trial were not designed or powered to assess variation in treatment effect by comorbidity, and relatively few trial participants had >3 comorbidities.” Page 2 lines 53-55

To the limitations section:

“Furthermore, the individual trials were neither designed not powered to measure comorbidity treatment interactions. Specifically, the higher levels of comorbidity observed in clinical practice (e.g. 5 or more comorbidities) are rare within the trial participants. This reduces the likelihood of detecting a comorbidity-treatment interaction were one to exist. Therefore, while our results are consistent with there being no comorbidity-treatment interactions, this should be interpreted within the range of comorbidities, index conditions and treatment comparisons that are presented.” Page 17 lines 443-449

And the conclusion:

“Nonetheless, comorbidity is under-represented in trials, especially at higher levels often seen in clinical practice, and in these contexts the applicability of trial effect estimates need to be carefully considered.” Page 18 lines 525-527

Reviewer comment:

3. What is the outcome model in the treatment*comorbidity interaction problem, within each trial? Does it also include age and sex interactions with the treatment? Can you write it out?

Author response:

This was described in the supplementary appendix:-

The treatment, age and sex models are written in full (please see section (i) Treatment, age and sex> IPD analysis) then the comorbidity count models were described  as follows (please see the section ii) comorbidity count) “Analyses for the comorbidity count-treatment interaction were similar except that the IPD analysis included two additional terms – comorbidity count and arm-comorbidity count interaction”. The comorbidity-treatment interaction models also contain the age- and sex- interaction terms and this is written out in full within the supplementary appendix (S1 File).

Reviewer comment:

4. I have a major concern with the manner in which treatment*comorbidity-count is modeled as a linear term, i.e. the impact of one-unit increase in comorbidity is the same whether it goes from 0 to 1 or from 5 to 6. This is a highly untenable assumption. I would recommend that the authors do another set of analyses where this variable is treated as a binary covariate: 2 or less versus 3 or more.

Author response:

We had originally run additional trial-level models with additional polynomial terms for comorbidity count^2 and arm-comorbidity count^2 interactions, but did not meta-analyse these as the linear terms were null. We felt this was justified as while claiming linear associations may be misleading, it would be unusual for a truly curvilinear association to be null if modelled using a linear term (outside contrived pedagogical examples). Nonetheless, we are very willing to meta-analyse comorbidity count and comorbidity-count squared. We have now done so, finding no evidence of an association:

<https://github.com/ChronicDiseaseEpi/hte_cm_public/blob/main/Outputs/como_sq_plot1.pdf>

<https://github.com/ChronicDiseaseEpi/hte_cm_public/blob/main/Outputs/como_sq_plot2.pdf>

<https://github.com/ChronicDiseaseEpi/hte_cm_public/blob/main/Outputs/como_sq_plot3.pdf>

https://github.com/ChronicDiseaseEpi/hte_cm_public/blob/main/Outputs/como_sq_plot4.pdf

Reviewer comment:

5. Were treatment*age and treatment*sex interactions included in the model where treatment*comorbidity was the focus?

Author response:

These were included in the trial-level models, but not in the meta-analysis models (please see response to question 1). Therefore, we were meta-analysing the comorbidity count-treatment interaction term having adjusted on age, sex and comorbidity as well as age-treatment and sex-treatment interactions.

Reviewer comment:

6. I was wondering why the main effect for treatment (“arm”) was not included in the meta-analysis of treatment*comorbidity interaction, while it was included in the meta-analysis of treatment*age and treatment*sex interactions. I would argue that the main effect ought to be included.

Author response:

The treatment effect, sex-treatment interaction and age-treatment interaction were included in the meta-analysis because all three parameters were of interest and as they came from the same model could be meta-analysed together. For the reasons outlined in the response to comment 5, we do not agree that “main effects” for interactions need to be included provided the coefficient being meta-analysed (eg the comorbidity-treatment interactions) come from trial-level models which have been appropriately adjusted for (as was the case here). Provided the main effects and other interactions are included in the trial level models, the interpretation of the meta-analysis for the coefficients is the same – it is the index condition/comparison level comorbidity-treatment interaction adjusted for age, sex, comorbidity as well as age-treatment and sex-treatment interactions. We do provide the full set of coefficients (and the full variance-covariance matrix) from each trial-level models. Therefore, these are available if other model coefficients are of interest to future researchers.

Reviewer comment:

7. I would also suggest sensitivity analyses for the prior parameters of the variance parameters: standard deviation of half-normal and the LKJ parameter for the correlation matrix. The paper should document how decreasing or increasing these parameters impacted the final meta-analytic estimates of the interaction effects.

Author response:

We have re-run the analysis after increasing the SD to 2 for the normal and half normal priors (was 1 previously) and increasing the LKJ shape prior to 2 (was 1). This made no difference to the results. We have added this plot and the underlying data to the accomanying repository:

<https://github.com/ChronicDiseaseEpi/hte_cm_public/blob/main/Outputs/sensitivity_prior.csv>

https://github.com/ChronicDiseaseEpi/hte_cm_public/blob/main/Outputs/sensitivity_plot.pdf

Reviewer comment:

8. I did not understand the logic behind generating an informative prior for conditions/treatment comparisons not included in our data. Can this be better explained?

Author response:

We apologise for not explaining this. We have added the following text (new text in bold):-

“In case other researchers wish to use the results of our models of treatment-covariate interactions to inform subsequent analyses as informative priors, we obtained summaries of the posterior predictions. We did so only for analysis (ii) for continuous outcomes. In order to provide a more general set of priors, we also predicted the comorbidity count-treatment interaction for treatment comparisons/conditions not included in our model by obtaining samples from the posteriors. **The latter are provided to allow researchers to conduct Bayesian analyses or probabilistic sensitivity analyses if studying conditions/treatment comparisons not included in our modelling as this represents a prediction for an unobserved index condition/treatment comparison (albeit one which is assumed to be exchangeable with the conditions/treatment comparisons included in the current analysis).”** New text in page 8 lines 272-277

Reviewer comment:

9. The authors state the following: “*We included the initial measurement among the covariates; this means that the coefficients for the remaining variables are interpretable as estimates for the change in the outcome measurement (ie final – base) per unit increment in the covariate*.”

This interpretation of the coefficients is not quite correct. It is the expected change in post-treatment response conditional on the pre-treatment response. It cannot be interpreted as

estimate of pre-post change for which one would have to model the conditional expectation of the change itself: E[final - base | covariates]

Author response:

Thank you, we have edited the text in the statistical appendix to the following:

“We included the initial measurement among the covariates; this means that the coefficients for the remaining variables are adjusted for pre-treatment response.”
