# Supplementary material for: Treatment effect modification due to comorbidity: Individual participant data meta-analyses of 120 randomised controlled trials
Source: PLoS Med. 2023 Jun 6;20(6):e1004176. doi: 10.1371/journal.pmed.1004176 (PMC10243630; doi:10.1371/journal.pmed.1004176)
Supplement: Attachment — Submitted filename: Response to editor comments.docx [file pmed.1004176.s009.docx]

Dear Dr Dodd,

Thank you for your decision letter indicating your intention to accept our manuscript for publication subject to the editorial comments. We have detailed our responses and changes to the text for each of these below.

Your sincerely

Dr Peter Hanlon, on behalf of the study authors

INDUSTRY SPONSORED TRIALS
The editorial team agree that it would be helpful to include a clear justification for including only industry-sponsored trial data and further discussion regarding the limitations of this approach. Please see the comments from the academic editor (below) in respect of the same, which we agree with.

Thank you for this comment. The reason for including only industry-sponsored trials is that these are the trials which are made available from the repositories from which we were able to access data. Access to other trials would have required separate data-sharing agreements with the primary data holders. We have edited the methods section making clear that the selection of industry-sponsored trials was pragmatic (driven by the availability of data) rather than a specific rationale for focusing the analysis on industry-sponsored trials alone. We have also discussed this as a limitation in the discussion section in response the comments from the academic editor below.

Changes to methods section:

“Eligibility criteria were ~~industry-sponsored~~ RCTs for one of the index conditions” (Page 6 line 155)

“These repositories facilitate sharing of industry-sponsored trial data with third-party researchers.” (Page 6 lines 162-163)

COMMENTS FROM THE ACADEMIC EDITOR
My initial assessment is that this is an interesting and important topic using a rather unique and hard to obtain group of data sets.

Some important caveats that the authors should emphasize are:
1) As this is industry sponsored trials, there may be some risk that these trials are not representative of the full range of trials -- designed with very tight exclusion/inclusion criteria or in other ways to boost the chance of getting a positive, significant finding in a desired direction and with very high and consistent compliance (more so than in other settings). This may compress the possibility of effect modification being detected BUT this does not mean that in other types of trials and studies the possibility of substantial effect modification still exists. Would be good to hear the authors' thoughts on this in the Discussion.

Thank you. We have added the following text to the discussion section:

“Fourth, while we include a large number of trials across a range of index conditions, this is not a representative sample in terms of the larger body of trials. Specifically, there were all industry-sponsored trials (as the CSDR and YODA repositories only held industry-sponsored trial data for the conditions of interest). Furthermore, not all sponsors share data in this way nor do sponsors share data for all trials conducted. We have previously demonstrated that these trials were similar to the wider body of industry-sponsored trials in terms of characteristics such as size, phase and significance of the primary outcome [4]. However, it is possible that my selecting only industry-sponsored trials inclusion criteria and selection processes may be more restrictive than for other trials. This means that, while we did not detect any evidence of treatment effect modification by comorbidity, it cannot be assumed to be absent particularly in other trials which may be more pragmatic or have less restrictive selection criteria.” (Page 17 lines 465-475)

2) Null trials. If the main effect is null (or very small) it is probably more likely that there will be no (detectable) effect modification. Meta-analyzing effect modification including studies w/o a main effect may not be how we standardly think about effect modification. Would be good to hear the authors' thoughts on this in the Discussion

We have added the following:

“Also, for some conditions and indications, the main effects were small or included the null. Where this was the case, the chances of detecting treatment effect modification are lower.” (Page 17 lines 459-461)

TITLE
Thank you for modifying your title, please remove 'industry-sponsored' suggest instead “Treatment effect modification due to comorbidity: An individual participant data meta-analysis of 126 randomised controlled trials” or something similar

Thank you, we have edited the title as suggested

INTRODUCTION
Line 131 “…byi)…” suggest “…by i)…”

Thank you for highlighting this. We have edited this error.

TABLES
Table 2: what to the asterisks refer to, it wasn’t entirely clear upon review, apologies if I have missed it, please clearly define. Row number 4 (age-treatment interaction), to improve accessibility to the reader please ensure the upper and lower bounds of CrIs are on a single line.

We have added the following to the footnote: “* indicates where the 95% credible interval does not include the null.” And have formatted the table as suggested so that the credible intervals on row 4 are on the same line.

Table 3: the footnote is labelled “Table 1” (line 388) please revise. Please define LMWH in the footnote.

Thank you. We have removed “Table 1” from the footnote and added the abbreviation definition as requested.

Line 548: please remove the competing interest statement from the main manuscript and include only in the manuscript submission form when you re-submit your manuscript, it will be compiled as metadata.

We have removed this.

REFERENCES
For intext reference callouts, please remove spaces from between different citations, for example line 89 “health priority [1, 2]…” should read “health priority [1,2]..” please check and amend throughout.

Thank you. We have edited the referencing throughout.

SOCIAL MEDIA
To help us extend the reach of your research, please provide any Twitter handle(s) that would be appropriate to tag, including your own, your coauthors’, your institution, funder, or lab. Please detail any handles you wish to be included when we tweet this paper, in the manuscript submission form when you re-submit the manuscript.

Thank you. We have included these on the submission form.
